# A perspective on conformational control of electron transfer in nitric oxide synthases

**DOI:** 10.1016/j.niox.2016.09.002

**Published:** 2017-02-28

**Authors:** Tobias M. Hedison, Sam Hay, Nigel S. Scrutton

**Affiliations:** Manchester Institute of Biotechnology, The University of Manchester, Manchester, United Kingdom

**Keywords:** Nitric oxide synthase, Cytochrome P450 reductase, Diflavin oxidoreductase, Fluorescence, Protein dynamics, NOS, nitric oxide synthase, CPR, cytochrome P450 reductase, CaM, calmodulin, MSR, methionine synthase reductase, P450 BM3, cytochrome P450 BM3, FAD, flavin adenosine dinucleotide, FMN, flavin mononucleotide, FRET, Förster resonance energy transfer

## Abstract

This perspective reviews single molecule and ensemble fluorescence spectroscopy studies of the three tissue specific nitric oxide synthase (NOS) isoenzymes and the related diflavin oxidoreductase cytochrome P450 reductase. The focus is on the role of protein dynamics and the protein conformational landscape and we discuss how recent fluorescence-based studies have helped in illustrating how the nature of the NOS conformational landscape relates to enzyme turnover and catalysis.

## Highlights

• The catalytic mechanism of NOS and related diflavin oxidoreductase has been extensively studied.
• Numerous studies have shown that NOS electron transfer chemistry is ‘gated’ by protein domain motion.
• Time-resolved fluorescence spectroscopy is a valuable tool for the study of NOS and related diflavin oxidoreductase dynamics.

## 1 Introduction

**Fig. 1:**
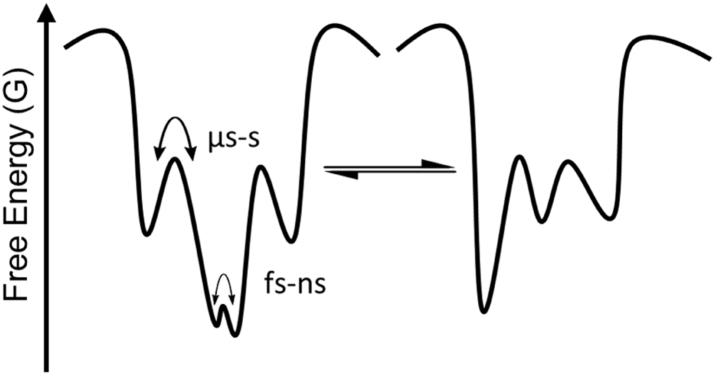
Simplified two dimensional depiction of a multidimensional conformational landscape of a protein molecule. The ‘valley’ features of the landscape represent (quasi)stable conformational states that interconvert via higher energy barriers or ‘hills’ [10], [11]. Temperature, pressure, mutagenesis, protein-protein interactions, reaction chemistry and ligand/inhibitor binding all influence the conformational landscape of a protein molecule (see text).

Many experimental and computational techniques have shown the importance of protein conformational change in both cell signalling [1], [2], [3], [4] and enzyme catalysis [5], [6], [7], [8], [9]. It is now commonly believed that X-ray crystallography-derived structural data, although valuable, is insufficient in describing the function of many proteins. This has led to the notion that the structure-function dogma should be expanded to encompass protein dynamics (structure-dynamics-function relationship) [10]. The dynamic profile of a protein can be thought of as a multidimensional conformational landscape, which comprises of ‘hill’ and ‘valley’ features representing high and low energy protein sub-states, respectively (Fig. 1) [10], [11]. These population sub-states can be easily perturbed, but also voluntarily controlled, by mutagenesis, temperature, pressure, protein-protein interaction, redox chemistry and ligand/inhibitor binding [10].

The field of protein dynamics is gaining increasing attention, as greater insight into protein function is required, in order to be able to develop target-directed pharmaceuticals [12], [13] and to rationally design enzymes for bio-catalytic purposes [14], [15]. However, the study of conformational changes associated with enzyme turnover is challenging, as dynamics occur over a broad range of time and distance scales, from sub-Ångstrom localised vibrations (femtoseconds) to large domain reorganisation (seconds) [9], [16].

**Fig. 2:**
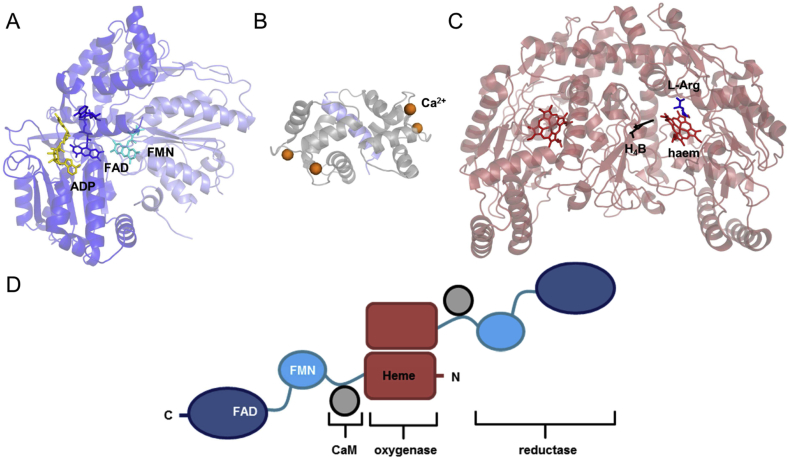
Structure and molecular architecture of NOS. A) The structure of the NADP^+^-bound neuronal NOS diflavin reductase domain (PDB ID 1TLL). B) The structure of holo-CaM bound to a nNOS-peptide (PDB ID 2O60). C) The crystal structure of the nNOS oxygenase domain dimer (PDB ID 1ZVL). CaM binds between the oxygenase and reductase domains of NOS. D) Structural organisation of the functional NOS dimer. The NOS FAD and FMN binding domains are shown in dark blue and light blue, respectively. CaM is shown in grey and the NOS oxygenase domain is shown in red.

Nitric oxide synthase (NOS) is proposed to make defined conformational changes during catalysis. NOS produces the small molecule nitric oxide (NO), which has a broad range of physiological roles from vasodilation to neurotransmission [17]. The three tissue specific NOS isoenzymes are homodimers that function by transferring electrons, which originate from NADPH, to the catalytic NOS haem porphyrin centre. Each NOS monomer is made up of *i)* a C-terminal reductase domain, comprising discrete FAD/NADP(H) and FMN binding domains, *ii)* an N-terminal oxygenase domain, which contains a tightly bound haem B and a tetrahydrobiopterin (H_4_B) molecule and *iii)* a binding site for calmodulin (CaM), which links the reductase and oxygenase domains (Fig. 2).

NOS is believed to shuttle between the so called ‘input’ and ‘output’ conformational states, which have different orientations of the reductase and oxygenase domains [18]. These two states are thought to be functionally relevant in ‘gating’ the precise flux of electrons from NADPH to the catalytic haem centre. In recent years there has been a variety of spectroscopic approaches used to probe NOS conformational change. Much of these data are summarised in recently published review articles on the general biochemistry and biophysics of NOS [19], [20], [21], [22], [23], [24], [25]. Herein, as an alternative perspective, we present how recently published fluorescence spectroscopic data have helped in illustrating the nature of the NOS conformational landscape related to catalysis. We offer an overview of the information gathered from single molecule and ensemble fluorescence spectroscopy studies for the three tissue specific NOS isoenzymes and also for the related diflavin oxidoreductase cytochrome P450 reductase (CPR).

## 2 Structure of the NOS isoenzymes

Despite the lack of an atomistic structure of full length NOS, the X-ray crystal structures of the individual NOS domains [18], [26], [27], [28], [29], [30], along with recently published cryo-EM data [31], [32], [33], have helped to illustrate the structural organisation of this enzyme (Fig. 2). The three tissue-specific NOS isoenzymes are homodimeric proteins which bind and are activated by CaM. Each NOS reductase domain contains distinct FAD and FMN binding subdomains (Fig. 2A) and this domain is homologous to CPR, a microsomal membrane-bound diflavin oxidoreductase, which transfers electrons to a multitude of partner proteins, e.g. cognate cytochrome P450 enzymes (CYPs) [34], [35]. Other members of the diflavin oxidoreductase family include methionine synthase reductase (MSR), a key enzyme in folate and methionine metabolism [36] and the reductase domain of the bacterial CYP cytochrome P450 BM3 (P450 BM3) [37].

The NOS reductase and oxygenase domains are connected by a CaM binding site. All three NOS isoforms bind to CaM, which is essential for NOS catalysis. However, the binding between both the constitutive NOS (cNOS) proteins [neuronal NOS (nNOS) and endothelial NOS (eNOS)] and CaM is Ca^2+^ dependent and reversible, while inducible NOS (iNOS)-CaM interactions occur regardless of intracellular calcium concentrations [20].

Many spectroscopic studies have shown the flux of electrons in NOS isoenzymes occurs from NADPH, through FAD and FMN cofactors, to the catalytic haem centre where NO is produced (see below). However, based on crystallographic data of the isolated NOS reductase domain in complex with NADP^+^, where the FAD and FMN cofactors are in proximity, the electron transfer from reductase to oxygenase domains is thought not to be possible, due to the occluded location of the FMN [18]. This structure suggests that a large scale ‘shuttling’ of the NOS FMN domain (over ∼70 Å), between the FAD and haem domains, is required for intra-NOS electron transfer. These types of large-scale domain dynamics have been observed at atomistic detail in variants of CPR using X-ray crystallographic techniques [38], [39], [40]. Moreover, this conformational control has recently been detected also in NOS using cryo-electron microscopy (cryo-EM), which has provided low resolution information on the molecular architecture of the protein [31], [32], [33].

## 3 Reaction mechanism of NOS

**Scheme 1:**
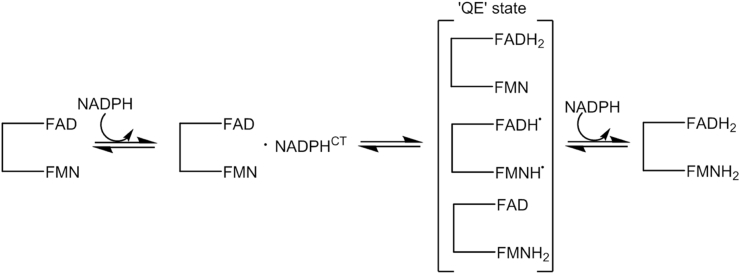
Simplified reductive half reaction of nitric oxide synthase and related diflavin oxidoreductases (see text for more details).

Due to the characteristic flavin and haem absorbance features, many UV–Vis based spectroscopic approaches, from conventional stopped-flow techniques [41], [42], [43], [44] to novel laser flash photolysis [45], [46], [47], [48], [49], have been used to probe NOS and related diflavin oxidoreductase redox chemistry. Flavin reduction is typically monitored in the stopped-flow by rapidly mixing the enzyme with NADPH and following the quenching of the ∼450 nm oxidised flavin feature or the growth and decay of the semiquinone species [42], [43], [50]. Due to the multiphasic nature of the stopped-flow traces for NOS reduction, the assignment of chemical steps to individual phases observed in the stopped-flow has not been possible [42], [43]. However, Scheme 1 shows the simplified mechanism for NADPH-driven flavin reduction, which has been advanced for NOS [42], [43] and is conceptually similar to that described for other related diflavin oxidoreductases [50]. Upon binding of NADPH to NOS, a hydride anion is transferred from the C4 position of the coenzyme to the N5 position of the oxidised NOS bound FAD cofactor. Following FAD reduction, reducing equivalents are transferred from the FAD hydroquinone to FMN, yielding the so called ‘quasi-equilibrium’ (QE) state - a thermodynamic equilibrium where electrons partition between the FAD and FMN cofactors. In the QE state, three predominant redox states are present (FAD hydroquinone and FMN oxidised; FAD semiquinone and FMN semiquinone; FAD oxidised and FMN hydroquinone). Following QE state formation, a second NADPH coenzyme binds to NOS and drives the equilibrium towards the FAD and FMN hydroquinone species.

The role of CaM during flavin reduction has been investigated. In full length nNOS [43], and in studies of isolated nNOS reductase domain [42], the binding of CaM has only small/marginal effects on the observed rates of interflavin electron transfer. However, CaM binding does alter the mid-point potential of NOS bound flavin [51], which will perturb the QE state and likely contributes to stimulated rates of cytochrome *c* reduction in steady-state assays in the presence of CaM [41], [42], [43], [44].

The chemistry catalysed by diflavin oxidoreductases diverges to some extent after FMN reduction. CPR and MSR transfer reducing equivalents from FMN to their respective partner proteins, while NOS and cytochrome P450 BM3 (P450 BM3) shuttle electrons from reduced FMN to their haem binding oxygenase domain. For NOS catalysis, the transfer of electrons from reduced FMN to haem is thought to be cross-monomer [52], [53] and requires the presence of CaM [54]. This FMN to haem electron transfer is difficult to study by stopped-flow based methods due to the slow rate of electron delivery along with the complexity of transients recorded. Thus, novel laser flash photolysis approaches, which can rapidly inject electrons into complex redox systems, have been used to track NOS FMN to haem electron transfer. In particular, studies of FMN to haem electron transfer using laser flash photolysis of carbon monoxide dissociation on partially reduced NOS have been used to access the dynamics and the chemistry catalysed by the enzyme. These measurements were initially performed on the truncated NOSoxyFMN construct [46], [47] and then subsequently on the full length NOS enzyme [45], [48], [49]. Discrepancies between the rates observed between these two forms of NOS constructs demonstrated that shuttling between NOS input and output states (dynamic interconversion) is rate limiting in NOS catalysis, providing evidence for a role of protein dynamics in gating NOS catalysis.

**Scheme 2:**
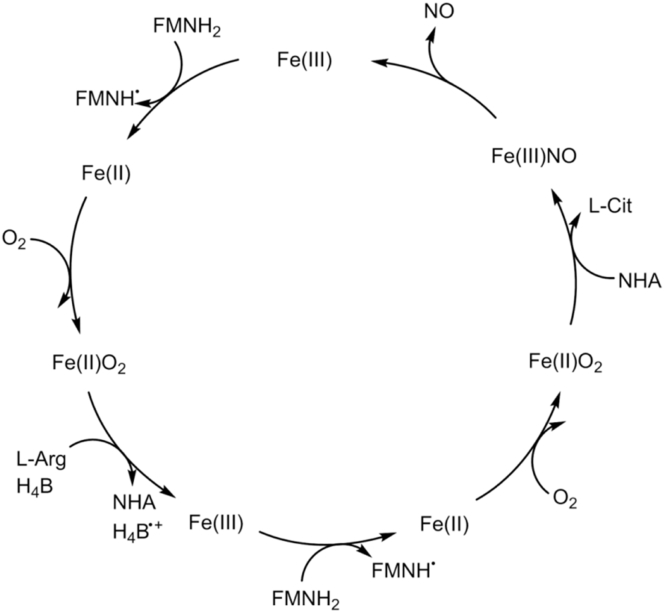
The sequential oxidation of l-arginine (L-Arg) into NO and l-citrulline (L-Cit) catalysed by NOS. Initially L-Arg is oxidised to N-hydroxy-l-arginine (NHA) which is further oxidised to L-Cit and NO. Electrons transfer from the NOS FMN hydroquinone to the NOS oxygenase domain is thought to be cross dimer and only occurs when CaM is bound.

After sequential electron transfer from FMN to haem, the signalling molecule NO along with the amino acid l-citrulline is produced at the NOS oxygenase domain, from l-arginine and molecular oxygen (Scheme 2) [55].

## 4 Domain dynamics of nitric oxide synthase (NOS)

### 4.1 Probing NOS dynamics using intrinsic flavin fluorescence

Using fluorescence to probe changes in the microenvironment of flavin chromophores (FAD and FMN), which are non-covalently bound to NOS, has proven to be a robust method of detecting conformational change in diflavin oxidoreductases [56]. Gachhui et al. [57] were the first to investigate NOS conformational change by observing FAD and FMN fluorescence steady state emission. The authors showed binding of Ca^2+^/CaM to the nNOS reductase protein caused an increase in flavin emission at ∼530 nm, when absorbance features of isoalloxazines were excited [57]. These CaM-dependent alterations in flavin emission have been observed in a range of experiments and are attributed to an increase in solvent exposure of the NOS FMN cofactor upon binding to CaM [51], [58], [59]. NADP^+^ binding has the opposite effect on NOS flavin fluorescence [51]. These data suggest that CaM binding promotes conformational freedom of the NOS FMN domain, while coenzyme binding hinders this mobility. This basic idea is consistent with a number of reports that suggest an auto-inhibitory loop and a C-terminal tail region of NOS make contact with the NADP(H) binding site [44], [60], [61], [62], [63]. In cNOS isoforms this interaction between coenzyme and the protein prevents movement of the FAD and FMN domains, fixing the protein in the ‘input’ state. When CaM binds to cNOS, this interaction is disrupted, enabling conformational freedom of the FMN domain. Overall, these steady-state fluorescence measurements have been useful in detecting how mutagenesis alters the conformational landscape of the enzyme, as well as showing that shuttling of the FMN domain between the ‘input’ and ‘output’ states limits catalysis (e.g. as demonstrated using solvent viscosity studies) [58], [59].

The study of NOS by flavin fluorescence lifetime decay measurements offers additional information on protein conformational change over steady state fluorescence emission measurements. Flavin lifetime measurements were first used to study the neuronal NOS (nNOS) holoenzyme. These studies showed that the recorded flavin fluorescence decay fits to a multiexponential model, indicating the presence of multiple flavin microenviroments (conformations) which do not interconvert on the fluorescence timescale [64]. Both cofactors (H_4_B and haem) and CaM/Ca^2+^ dependent changes in the fluorescence lifetime decays were observed, giving clues into how the nNOS oxygenase and reductase domains fold back onto one another and into the role of CaM in shuttling the FMN domain [64]. Cryo-EM structure of the full length enzyme [31], [32], [33] has recently confirmed what was inferred from this fluorescence study thereby demonstrating the capabilities of fluorescence spectroscopy to detect dynamic changes.

NOS fluorescence lifetimes were recently revisited by Salerno and co-workers [65], [66]. The authors probed the differences between the iNOS holoenzyme and the iNOSoxyFMN complex, allowing them to assign specific phases seen in the multiexponential fluorescence lifetime decay to certain conformational states related to NOS catalysis [66]. This was taken further in subsequent work published by the same authors on the nNOS isoform [65]. Combined, these studies with iNOS and nNOS showed the presence of three major conformational states of the NOS enzymes. Two of these states are the well-defined ‘input’ (lifetime of ∼100 ps) and ‘output’ (lifetime of ∼1 ns) states, where the FMN domain is closer to the FAD domain or haem domain, respectively. The third state detected was defined as an intermediate state (‘open’ state with a lifetime of ∼4.3 ns), where the FMN domain is distanced from the FAD and the haem domains. This ‘open’ state was reported to be heterogeneous (tail of fluorescence decays could fit to both single and multiple exponential functions) [65] and similar to recently published cryo-EM [31], [32], [33] and EPR [67], [68], [69] studies. These studies therefore reveal the complex and rugged nature of the NOS conformational landscape, which is also altered by CaM binding.

### 4.2 Probing NOS dynamics using external CaM-bound fluorophore fluorescence

The commercial availability of extrinsic cysteine and lysine-binding fluorophores has significantly contributed towards the understanding of protein conformational change. Labelling a protein with fluorophores in specific locations enables one to probe conformational change by a number of fluorescence based methods. However, unlike CPR [34], [35], NOS has multiple solvent exposed cysteine and lysine residues, some of which are crucial for structural integrity. Therefore, it is challenging to site-specifically label NOS with extrinsic fluorophores [70]. Alternatively, wild-type CaM does not have any innate cysteine residues, so site-specific labelling of CaM variants offers a tractable approach to the study of CaM-NOS dynamics by fluorescence spectroscopy.

**Fig. 3:**
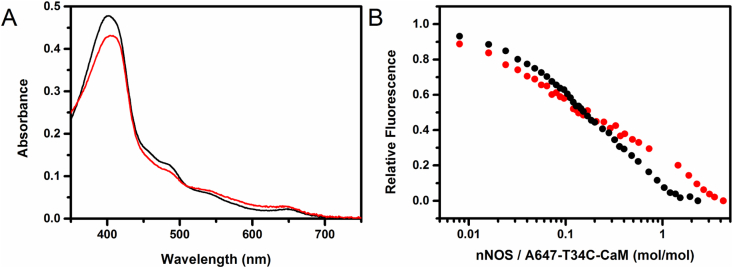
Redox-dependent binding of CaM to nNOS. A) shows the UV–visible absorbance spectra of oxidised (black) and dithionite-reduced (red) nNOS (∼5 μM). The dithionite reduced NOS was shown to be stable for over 2 h under air and the absorbance is not perturbed by the addition of a 5-fold excess of T34C-CaM (data not shown). B) shows the titration of 5 nM A647-T34C-CaM with oxidised (black) and dithionite-reduced (red) nNOS monitored by quenching of the fluorescence emission from the A647 bound to CaM. All materials were of analytic grade and purchased from Sigma-Aldrich, except Alexa Fluor 647 C2 maleimide (A647)m which was purchased from Thermofisher scientific. Both recombinant rat neuronal nitric oxide synthase and the T34C-CaM variant were expressed and purified as previously described [43], [69], [78], [79]. Purified nNOS was oxidised with ferricyanide and passed down a desalting column to remove excess oxidising agent. To reduce the oxidised nNOS, excess sodium dithionite mixed with the oxidised for of nNOS, which was subsequently passed down a desalting column equilibrated with the buffering solution. A647 maleimide was bound to T34C-CaM in the dark using previously published protocols [43]. Fluorescence measurements were made with an Edinburgh Instruments (Livingston, UK) FLS920 fluorometer. Emission spectra were taken using 5 nm excitation and 5 nm emission slit-widths in 1 mL fluorescent quartz cells (Starna Scientific Ltd, Hainault, UK) with a 10 mm excitation path length. Data were collected at room temperature in 40 mM HEPES (pH 7.6), 150 mM NaCl,1 mM CaCl_2_ and 10% glycerol. Under these conditions, no fluorophore photo-bleaching was observed.

**Fig. 4:**
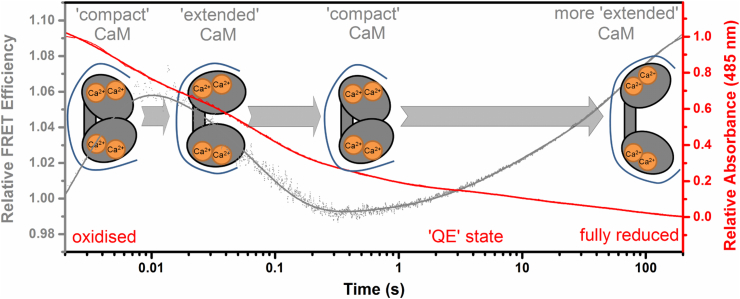
nNOS-bound CaM dynamics and reaction chemistry are kinetically coupled. The graph shows the transient reaction chemistry of nNOS (relative absorbance, red) and dynamics of nNOS-bound CaM (relative FRET efficiency, grey) recorded when mixing excess NADPH with nNOS (bound to CaM), under pseudo first order conditions. The schematic presented here shows the temporal resolved dynamics of nNOS-bound CaM when nNOS is reduced with NADPH. This figure is adapted in part from Ref. [43].

A number of well characterised CaM Cys knock-in variants have been created that can be labelled with extrinsic fluorophores [43], [68], [71], [72], [73], [74], [75]. These variants, which have no reported effect on NOS turnover, have been used to study the kinetics of CaM-NOS association as well as deciphering the antiparallel-orientation (N-terminal of NOS binds to C-terminal of CaM and *vice versa*) and the compact configuration (short distance between C- and N-terminal) that CaM adopts when bound to NOS enzymes [74], [75]. However, much of this work has focused on the use of truncated versions of NOS and there has been little focus on the interactions between full length NOS and CaM. In an attempt to assess any differences in the redox-dependent association of CaM and nNOS, we have labelled one of these commonly used CaM variants (T34C-CaM) with the cysteine binding Alexa-647 maleimide fluorophore. In this study the T34C-CaM bound Alexa-647 maleimide fluorescence was seen to quench upon binding to nNOS, likely due to Förster resonance energy transfer (FRET) from the fluorophore to chromophore(s) in nNOS. Fig. 3 shows the titrations of Alexa-647 labelled T34C-CaM with both oxidised and aerobic dithionite-reduced nNOS. Due to the complex nature of the tight binding between CaM and nNOS, as well as the presence of different NOS-CaM binding states (previously observed by EPR [68], fluorescence lifetime and single-molecule studies [72]), dissociation constants (*K*_d_) values were not calculated for the CaM-nNOS interaction. However, by inspection of the two different CaM-nNOS titration curves, a clear variation can be observed. These data provide compelling evidence that redox-chemistry drives conformational changes in the nNOS enzyme, which in turn has a knock-on effect on CaM binding. These redox-dependent conformational changes have been seen in the related diflavin oxidoreductase CPR. When CPR is reduced to two- or four-electron reduced forms, the FAD and FMN containing domains move relative to one another, and similar behaviour could explain why the interactions between NOS and CaM are altered by redox chemistry [76], [77]. We have recently probed the redox-dependent conformational changes of nNOS-bound CaM by using a novel stopped-flow FRET technique [43], which was initially developed to track temporally-resolved changes in CPR domain organisation (see section below [34], [35]). We subsequently labelled a double cysteine containing CaM variant (T34C/T110C-CaM) with both donor and acceptor fluorophores (Alexa 555 and Alexa 647). By monitoring time-resolved changes in fluorescence emission by stopped-flow spectroscopy, we were able to detect defined conformational changes in the CaM-NOS complex and showed that CaM dynamics are kinetically coupled to key mechanistic steps during nNOS turnover. These two fluorescence studies show that redox chemistry, along with cofactor binding, drives conformational change in the NOS enzymes, and is essential for ‘gating’ electron transfer from NADPH to the NOS catalytic haem centre (see Fig. 4).

### 4.3 Probing NOS dynamics using external NOS-bound fluorphore fluorescence

As discussed above, wild-type NOS domain dynamics are difficult to probe using FRET-based experiments due to the high number of potential fluorophore labelling sites. Recently, He et al. constructed a ‘Cys-lite’ variant of the NOS reductase domain [70] that is unreactive towards maleimide fluorophores. This subsequently enabled these authors to further mutate the variant to allow the successful site-specifically labelling of NOS with fluorophores. Single molecule FRET measurements [70] were used to monitor the distances between the nNOS FAD and FMN binding domains, allowing the conformational landscape of NOS to be probed. The main finding from this study is that CaM alters the distribution of NOS conformational states, increasing the distances between the FAD and FMN binding domains as well as increasing the rate of conversion between protein sub-states and narrowing the distribution of NOS conformers [70]. These alterations in the NOS conformational landscape apparently enables the enzyme to form more productive geometries, increasing the flux of electrons from NADPH to the haem catalytic centre, as is evident by the stimulation of NOS catalytic activity by CaM [41], [43], [44].

**Fig. 5:**
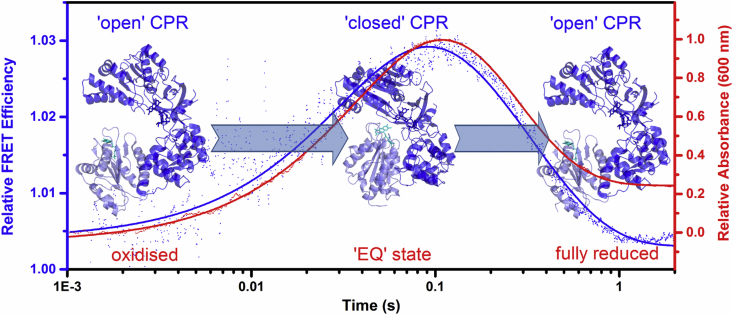
CPR domain dynamics and reaction chemistry are kinetically coupled. The graph shows the transient reaction chemistry (relative absorbance, red) and dynamics (relative FRET efficiency, blue) of CPR when mixing NADPH with the enzyme under pseudo first order conditions. The schematic shows the structures of the ‘open’ (PDB ID 3ES9) and ‘closed’ (PDB ID 1AMO) CPR structures with the FAD and FMN binding domains in dark blue and light blue, respectively. This figure is adapted in part from Ref. [34].

CPR has far fewer solvent exposed cysteine residues than NOS (3 cf. 14) and can be labelled with two extrinsic fluorophores [34]. Labelling of CPR with a mixture of donor and acceptor fluorophores allows the tracking of conformational changes that affect the FAD-FMN separation [34], [35] and we have used this approach to investigate single-turnover domain dynamics that occur during CPR catalysis. Coenzyme (NADP^+^) binding causes CPR to adopt a ‘closed’ conformation with shorter distances between the FAD and FMN cofactors [35] and stopped-flow experiments have illustrated that CPR undergoes defined conformational changes (‘opening’ and ‘closing’) during turnover, which appear to occur on the same timescale as redox chemistry (Fig. 5) [34], [35]. These time-resolved domain dynamics of CPR had not been detected prior to these measurements, and may play a role in gating electron transfers in CPR as well as in the NOS isoenzymes.

## 5 Conclusions

Various studies of NOS and related diflavin reductases now give a largely consistent picture where these enzymes exist in multiple conformational states that can be described by a rugged conformation landscape, which is perturbed by CaM and coenzyme binding, flavin (and possibly haem) redox state and also solvent environment (viscosity, ionic strength, etc.). Stopped-flow-based FRET studies of CPR and CaM-nNOS have recently begun to show how enzyme chemistry (flavin reduction) maps onto conformational changes in these enzymes and now allows a mapping between conformational and catalytic landscapes in diflavin oxidoreductases.
